# The cancer inflammation prognostic index is a valuable biomarker for predicting the survival of patients with stage I–III colorectal cancer

**DOI:** 10.1038/s41598-023-45550-0

**Published:** 2023-10-23

**Authors:** Hailun Xie, Lishuang Wei, Mingxiang Liu, Yanren Liang, Qiwen Wang, Shuangyi Tang, Jialiang Gan

**Affiliations:** 1 Department of Colorectal and Anal Surgery, The First Affiliated Hospital, Guangxi Medical University, 6 Shuangyong Road, Nanning, 530021 Guangxi People’s Republic of China; 2 Department of Geriatric Respiratory Disease Ward, The First Affiliated Hospital, Guangxi Medical University, Nanning, Guangxi People’s Republic of China; 3 Guangxi Key Laboratory of Enhanced Recovery After Surgery for Gastrointestinal Cancer, Nanning, Guangxi People’s Republic of China; 4 Department of Pharmacy, The First Affiliated Hospital, Guangxi Medical University, 6 Shuangyong Road, Nanning, 530021 Guangxi People’s Republic of China

**Keywords:** Cancer, Tumour biomarkers

## Abstract

This study aimed to assess the relationship between the Cancer-Inflammation Prognostic Index (CIPI) and disease-free survival (DFS) and overall survival (OS) in patients with stage I–III colorectal cancer (CRC). The relationship between the CIPI and survival was evaluated using restricted cubic splines. Survival curves were established using the Kaplan–Meier method and the log-rank test. Cox proportional hazards models were used to explore independent prognostic factors for CRC. Meaningful variables from the multivariate analysis were used to construct prognostic nomograms. The relationship between the CIPI values on a continuous scale and the risk of DFS/OS mortality was an inverted L-shape. Patients with a high CIPI had significantly lower DFS (53.0% vs. 68.5%, *p* < 0.001) and OS (55.5% vs. 71.7%, *p* < 0.001) than those with a low CIPI. The CIPI can also serve as an effective auxiliary tool to further distinguish the prognosis of patients with CRC at the same pathological stage, especially for stages II and III. After multivariate adjustment, a high CIPI was found to be an independent risk factor for DFS (HR 1.443, 95% CI 1.203–1.730, *p* < 0.001) and OS (HR 1.442, 95% CI 1.189–1.749, *p* < 0.001) in CRC patients. These nomograms have the advantage of integrating individual profiles, tumour characteristics, and serum inflammatory markers, providing favourable discrimination and calibration values. Compared with traditional TNM staging, nomograms have a better predictive performance. The CIPI is an effective and easy-to-use clinical tool for predicting the recurrence and overall mortality of patients with stage I–III CRC.

## Introduction

Colorectal cancer (CRC) is the third-most frequently diagnosed cancer globally, accounting for approximately 10.0% of all new cancers, and is considered the second-most common cause of cancer-related deaths, accounting for approximately 9.4% of all cancer-related deaths^1^. In China, CRC remains a major threat to human lives and health. The incidence and mortality of CRC rank second and fourth, respectively, among all cancers^2^. Despite recent improvements in multidisciplinary treatment approaches, including surgery, chemotherapy, and radiotherapy, CRC mortality rates remain high^3,4^. The disease progression and prognoses of patients with CRC are widely believed to be determined by pathological stage, marginal status, and specific histological and molecular characteristics^5,6^. Furthermore, CRC is a heterogeneous disease. Even among patients at the same stage, differences occur in outcomes and responses to treatment. Therefore, identifying readily available, convenient, practical, and preoperative biomarkers has tremendous clinical benefits for identifying patients at greater risk of a poor prognosis. Compared with traditional prognostic indicators, including tumour size, pathological stage, and perineural/vascular invasion, blood biomarkers have greater potential for predicting prognoses and guiding the treatment of CRC patients because of their easy accessibility and minimally invasive features.

Systemic inflammation is the most representative tumour–host interaction in cancer. In the process of tumour pathogenesis and development, activated pro-inflammatory cytokines and inflammatory cells promote the formation of new lymph and blood vessels, thus creating a tumour microenvironment conducive to the growth and differentiation of tumour cells. Systemic inflammation also destroys immune cell function, making tumour cells more prone to invasion and metastasis^7–9^. Increasing evidence suggests that the combination of blood-based systemic inflammation parameters is associated strongly with an adverse prognosis in patients with CRC^10–12^. Carcinoembryonic antigen (CEA) is the most acceptable and conventional tumour marker for CRC and is used widely for screening, predicting treatment response and survival, and detecting CRC recurrence^13–15^. Several studies have reported that high preoperative serum CEA levels are closely associated with a poor prognosis for patients with CRC^16–18^. However, serum CEA levels are not specific for CRC, and more than half of patients with CRC have serum CEA levels within the normal range. Therefore, other indicators are necessary for improving the ability of serum CEA levels to predict the prognoses of patients with CRC. Recently, a novel prognostic biomarker, the Cancer-Inflammation Prognostic Index (CIPI), combined with neutrophil, lymphocyte, and CEA levels, has been reported to be an effective predictor of the prognosis of patients with CRC^19^. Neutrophils and lymphocytes are simple and effective indicators associated with systemic inflammation and are valuable predictors of a poor prognosis in patients with CRC^20,21^. Serum CEA is a protein secreted from the tumour itself and is indicative of the tumour load. Therefore, the serum CIPI is a comprehensive integration of tumour–host interactions and tumour-related factors and has a wide range of potential applications in predicting the prognoses of patients with CRC.

At present, little research is available on the relationship between the CIPI and prognosis in CRC patients. In this study, we aimed to explore the value of the CIPI for predicting disease-free survival (DFS) and overall survival (OS) in patients with stage I–III CRC, using real-world data from a single centre. In addition, we constructed CIPI-based prognostic nomograms to provide a reference for the individualised monitoring of relapse risk and survival outcomes in patients with CRC.

## Materials and methods

### Study population

This retrospective study included patients with stage I–III CRC admitted to the First Affiliated Hospital of Guangxi Medical University between 2012 and 2015. Patients were selected according to the following inclusion criteria: (1) age > 18 years; (2) colorectal adenocarcinoma confirmed by histology or cytology; and (3) complete clinical, laboratory, and follow-up data. Patients were excluded according to the following criteria: (1) previous diagnosis of malignancy, (2) emergency surgery or non-curative excision, (3) having received neoadjuvant radiotherapy or chemotherapy, (4) there is clear clinical evidence of infection or inflammation, such as an elevated white blood cell greater than 15 × 10^9^/L, or a documented use of antibiotics or steroids in the medical records. and (5) taking anti-inflammatory medications. Ultimately, 1304 patients were included in the study. Informed consent was obtained from all eligible patients, and the study was approved by the Ethics Committee of the First Affiliated Hospital of Guangxi Medical University. All methods were carried out in accordance with the principles of the Declaration of Helsinki.

### Data collection and indicator definition

The following data were collected: sex, age, height, weight, comorbidities (hypertension and diabetes), T stage, N stage, TNM stage, tumour location (rectal and colon), tumour size, perineural/vascular invasion, macroscopic type, differentiation, postoperative radiotherapy, and postoperative chemotherapy. Peripheral venous blood was collected from all patients within 48 h after admission, prior to surgery, for complete blood counts and tumour markers analysis. Body mass index was defined as the weight (kg)/height-squared (m^2^). TNM staging was performed using AJCC Cancer Staging 8th Edition. The serum CIPI was defined as the CEA concentration (mg/L) × neutrophil count (10^9^/L)/lymphocyte count (10^9^/L). Other prognostic indicators include the prognostic nutrition index (PNI), neutrophil-to-lymphocyte ratio (NLR), platelet-to-lymphocyte ratio (PLR), and lymphocyte-to-monocyte ratio (LMR). PNI is defined as serum albumin (g/L) plus 5 times the lymphocyte count (× 10^9^/L). The neutrophil-to-lymphocyte ratio (NLR) is defined as the ratio of neutrophil counts (× 10^9^/L) to lymphocyte counts (× 10^9^/L). PLR is defined as the ratio of platelet counts (× 10^9^/L) to lymphocyte counts (× 10^9^/L). LMR is defined as the ratio of lymphocyte counts (× 10^9^/L) to monocyte counts (× 10^9^/L).

### Follow-up and outcome

Patients with CRC were followed up every 3 months for 2 years after surgery and every 6 months after that, with the last follow-up date being 31 July 2021. Follow-up included serological tests, plain abdominal radiography, abdominal computed tomography, and fibrous colonoscopy. DFS was defined as the period between radical resection and the first recurrence, death, or final follow-up date. OS was defined as the period between radical resection and the date of death or final follow-up.

### Statistical analysis

Categorical variables were expressed as frequency and proportion and compared using the chi-squared test. Continuous variables were expressed as mean ± standard deviation and analysed using Student’s t-test. The ability of the CIPI and other prognostic indicators to categorise disease status was evaluated using receiver operating characteristic (ROC) curves. The Youden-index was used to determine the optimal cut-off for a CIPI based on disease outcome. The relationships between the CIPI and survival were evaluated on a continuous scale with restricted cubic splines fitted for Cox proportional hazards models. Survival curves were established using the Kaplan–Meier method, and differences were analysed using the log-rank test. To identify independent prognostic factors, Cox proportional hazards models were used, and the corresponding hazard ratios (HRs) and 95% confidence intervals (CI) were calculated. Variables with *p* values < 0.05 in univariate analysis were entered into multivariate analysis using the backward condition method. In the multivariate Cox proportional hazards models, we used Schoenfeld residuals to assess the proportional hazards assumption. When none of the covariates showed statistical significance (*p* > 0.05), we can conclude that the model adheres to the proportional hazard’s assumption. Meaningful variables from the multivariate analysis were used to construct CIPI-based prognostic nomograms. We utilized the “rms” package to perform COX regression analysis for scoring values to each indicator. The predictive ability of these nomograms was evaluated using the consistency index (C-index) and a calibration curve. Time-dependent ROC and decision curve analysis (DCA) were used to compare the ability of the nomograms to predict prognoses with that of traditional TNM staging. A double-tailed *p* < 0.05 was considered statistically significant. Statistical analysis was performed using R 4.0.2 software (http://www.R-project.org).

### Ethical approval

Informed consent was obtained from all eligible patients, and the study was approved by the Ethics Committee of the First Affiliated Hospital of Guangxi Medical University (Registration number: NO.2022-KY-(043)).

### Consent to participate

Written informed consent from participants was required in accordance with local/national guidelines.

## Results

### Clinicopathological characteristics of patients based on the CIPI

Among the 1304 patients included in the study, the median age was 58.31 (± 13.00) years, including 821 (63.0%) males and 483 (37.0%) females. The median follow-up time was 67 (56–80) months. The differences in the demographic and clinicopathological characteristics of the patients are shown in Table S1. ROC curve analysis identified that the optimal threshold for predicting the output of CRC patients was 10.831, with a sensitivity of 0.647, specificity of 0.527 (Fig. S1), and area under the curve (AUC) of 0.617 (95% CI 0.585–0.649; *p* < 0.001). To simplify grouping and application, we determined that the recommended threshold for the CIPI was 11 for patients with stage I–III CRC, with a sensitivity of 0.651 and a specificity of 0.523.

The CIPI in all patients ranged from 0.18 to 16945.67, with a mean of 62.01 ± 591.78 and a median of 8.12. In this study, the median CIPI for CRC patients with recurrence was 10.93 (95% CI 4.88–46.87), while that for CRC patients without recurrence was 7.38 (95% CI 3.61–22.39) (Fig. S2A). Similarly, the median CIPI of CRC patients who died was much greater than that of non-dying CRC patients, namely 11.95 (95% CI 5.35–40.68) and 6.97 (95% CI 3.42–18.75), respectively (Fig. S2B).

A total of 762 CRC patients were identified as having a low CIPI (< 11), and 542 CRC patients were identified as having a high CIPI (≥ 11). We found that a high CIPI was significantly associated with male sex, hypertension, advanced T stage, advanced N stage, advanced TNM stage, colon cancer, larger tumour size, high neutrophil count, low lymphocyte count, and high CEA level. In addition, the overall mortality of patients in the high-CIPI group was 16.2% greater than that in the low-CIPI group; The length of hospitalisation of patients in the high-CIPI group was 1 day longer than that in the low-CIPI group; The hospitalisation cost of patients in the high-CIPI group was 2529.19 Yuan more than that of patients in the low-CIPI group (Table 1).

**Table 1:** The relationships between the CIPI and clinicopathological characteristics of patients with colorectal cancer.

Clinicopathological characteristics	CIPI		*P* value
	Low (n = 762)	High (n = 542)	
Sex(Man)	457 (60.0)	364 (67.2)	0.010
Age (mean (SD))	57.22 (13.15)	59.85 (12.63)	< 0.001
BMI (median [IQR])	22.15 (19.98, 24.61)	22.05 (20.09, 24.22)	0.507
Hypertension (Yes)	113 (14.8)	105 (19.4)	0.036
Diabetes (Yes)	44 (5.8)	38 (7.0)	0.429
T stage (T3–4)	494 (64.8)	446 (82.3)	< 0.001
N stage			< 0.001
N0	484 (63.5)	280 (51.7)	
N1	187 (24.5)	164 (30.3)	
N2	91 (11.9)	98 (18.1)	
TNM stage			< 0.001
Stage I	214 (28.1)	70 (12.9)	
Stage II	272 (35.7)	208 (38.4)	
Stage III	276 (36.2)	264 (48.7)	
Perineural invasion (Yes)	66 (8.7)	56 (10.3)	0.355
Vascular invasion (Yes)	109 (14.3)	99 (18.3)	0.065
Macroscopic type			0.380
Protrude type	230 (30.2)	145 (26.8)	
Infiltrating type	60 (7.9)	42 (7.7)	
Ulcerative type	472 (61.9)	355 (65.5)	
Differentiation (Poor)	93 (12.2)	75 (13.8)	0.433
Tumor location (Rectal)	426 (55.9)	261 (48.2)	0.007
Tumor size (median [IQR])	4.00 (3.00, 5.00)	5.00 (4.00, 6.00)	< 0.001
CEA (High)	59 (7.7)	435 (80.3)	< 0.001
Neutrophil	3.48 (2.78, 4.34)	4.42 (3.48, 6.01)	< 0.001
Lymphocyte	1.87 (1.54, 2.33)	1.57 (1.19, 1.99)	< 0.001
Radiotherapy (Yes)	84 (11.0)	40 (7.4)	0.034
Chemotherapy (Yes)	349 (45.8)	232 (42.8)	0.310
Death (Yes)	216 (28.3)	241 (44.5)	< 0.001
Length of stay (median [IQR])	16.00 (11.00, 20.00)	17.00 (12.00, 21.00)	0.017
Hospitalization cost (median [IQR])	48,702.60 (44,316.49, 54,258.84)	51,231.79 (45,084.82, 58,571.19)	< 0.001

*CRC* colorectal cancer; *BMI* body mass index; *CIPI* cancer-inflammation prognostic index.

### Comparison with other prognostic markers

**Figure 1:**
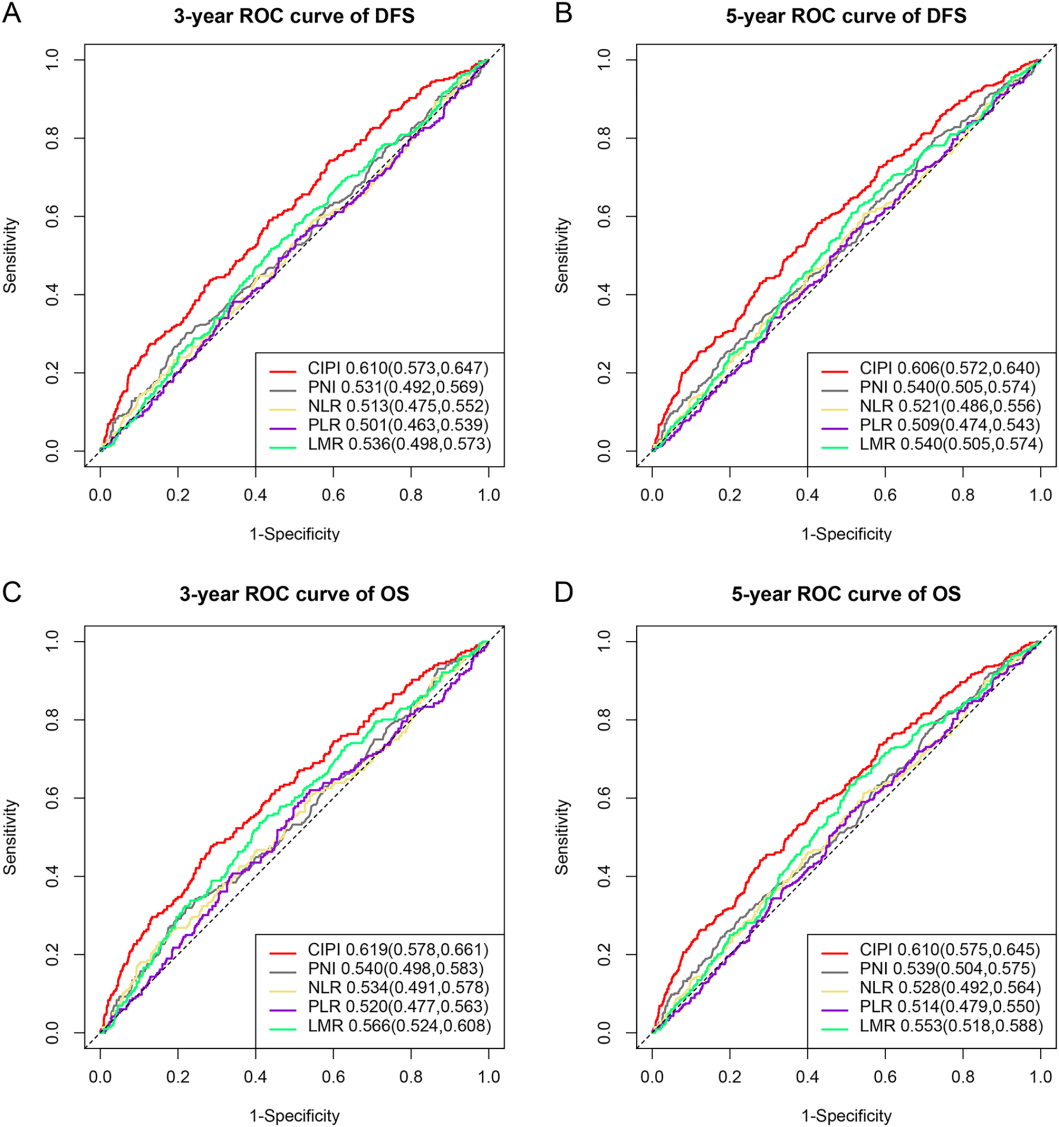
Comparison with other prognostic indicators. *Notes* (**A**) 3 years ROC curve of DFS; (**B**) 5 years ROC curve of DFS; (**C**) 3 years ROC curve of OS; (**D**) 5 years ROC curve of OS.

In order to compare the prognostic ability of CIPI with other prognostic markers for predicting the prognosis of CRC patients, we plotted ROC curves and calculated their AUCs. For 3-year DFS, the AUC of the CIPI curve was superior to those of PNI, NLR, PLR, and LMR (0.610 vs. 0.531 vs. 0.513 vs. 0.501 vs. 0.536) (Fig. 1A). Similarly, for 5-year DFS, the AUC of the CIPI curve was superior to those of PNI, NLR, PLR, and LMR (0.606 vs. 0.540 vs. 0.521 vs. 0.509 vs. 0.540) (Fig. 1B). In predicting 3 years OS (0.619 vs. 0.540 vs. 0.534 vs. 0.520 vs. 0.566) (Fig. 1C) and 5 years OS (0.610 vs. 0.539 vs. 0.528 vs. 0.514 vs. 0.553) (Fig. 1D), CIPI showed better prognostic prediction efficiency compared to other prognostic markers.

### Kaplan–Meier survival curve of DFS and OS

**Figure 2:**
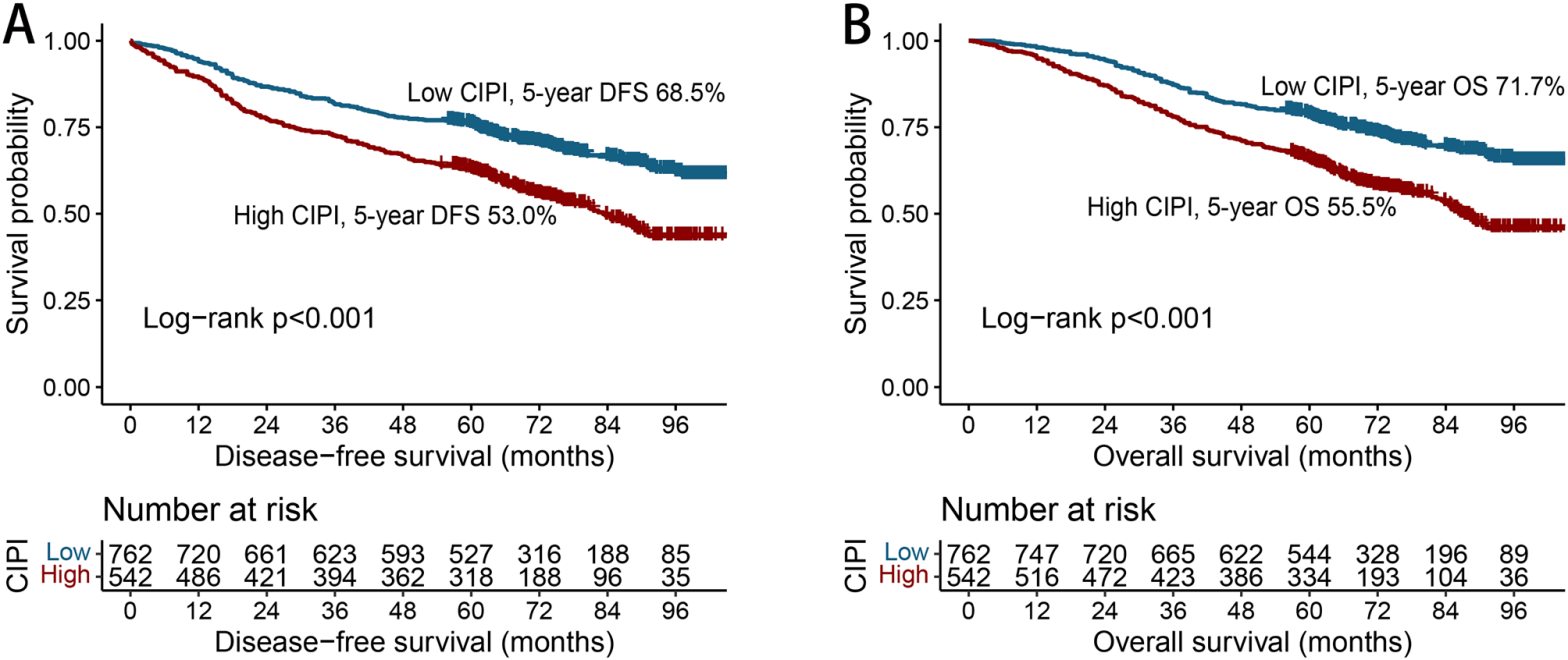
Kaplan–Meier curve of CIPI in patients with colorectal cancer. *Notes* (**A**) Disease-free survival; (**B**) Overall survival.

A total of 308 patients (23.6%) had recurrence during the follow-up period, with a greater recurrence rate in the high-CIPI group than in the low-CIPI group (28.6% vs. 20.1%). The Kaplan–Meier survival curve showed that the 5-year DFS of CRC patients with a high CIPI was significantly less than that of CRC patients with a low CIPI (53.0% vs. 68.5%, *p* < 0.001) (Fig. 2A). In a subgroup analysis of TNM staging, the CIPI significantly stratified the prognosis of CRC patients at stage II (62.0% vs. 74.6%, *p* = 0.002) and stage III CRC (39.4% vs. 54.3%, *p* < 0.002). However, no significant difference was found for CRC patients at stage I (Fig. S3A–C).

A total of 457 patients (35.0%) died. The mortality rate in the high-CIPI group was 44.5%, which was much greater than that in the high-CIPI group (28.3%). Patients with a high CIPI had a significantly lower OS rate than those with a low CIPI (55.5% vs. 71.7%, *p* < 0.001) (Fig. 2B). The CIPI could not effectively differentiate the outcomes in patients with stage I disease (Fig. S3D). For stage II disease, the OS of patients with a high CIPI was significantly less than that of patients with a low CIPI (63.9% vs. 77.6%, *p* < 0.001) (Fig. S3E). For stage III disease, the CIPI could also significantly stratify the prognoses of patients (42.8% vs. 57.6%, *p* < 0.006) (Fig. S3F).

### The relationship between the CIPI and clinical prognosis

**Figure 3:**
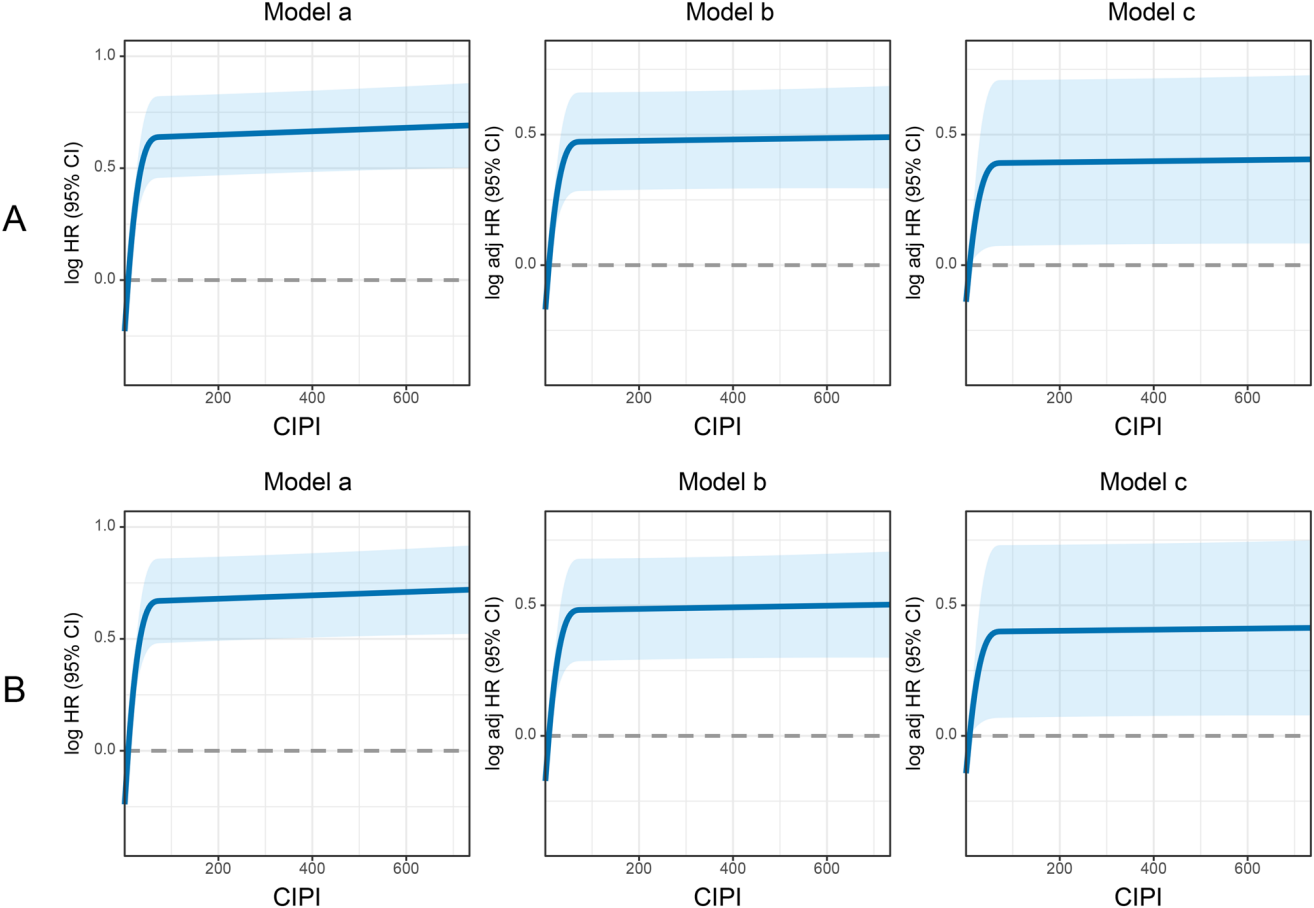
The association between CIPI and survival in patients with colorectal cancer. *Notes* (**A**) Disease-free survival; (**B**) Overall survival. Model a: No adjusted. Model b: Adjusted for gender, age, and BMI. Model c: Adjusted for gender, age, BMI, hypertension, diabetes, T stage, N stage, tumor location, tumor size, perineural invasion, vascular invasion, macroscopic type, differentiation, radiotherapy, chemotherapy.

The relationship between the CIPI values on a continuous scale and the risk of DFS mortality had an inverted L-shape. High CIPI values were associated with an increased risk of DFS (Fig. 3A). This relationship was also found between CIPI values and the risk of OS mortality. As CIPI values increased, the OS mortality of patients with CRC gradually increased (Fig. 3B). A high CIPI was significantly associated with an increased risk of DFS mortality in the univariate analysis of DFS (HR 1.694, 95% CI 1.420–2.021, *p* < 0.001). After multivariate adjustment, a high CIPI was an independent risk factor for DFS (HR 1.443, 95% CI 1.203–1.730, *p* < 0.001) (Table 2). In univariate analysis of OS, a high CIPI was significantly associated with an increased risk of OS mortality (HR 1.780, 95% CI 1.481–2.139, *p* < 0.001). After multivariate adjustment, a high CIPI remained independently associated with the risk of poor OS mortality for patients with CRC (HR 1.442, 95% CI 1.189–1.749, *p* < 0.001) (Table 3). To investigate whether clinicopathological variables changed the effect of CIPI values on survival, we performed a multivariate subgroup analysis based on various clinical features. The results showed that a high CIPI was an independent risk factor for DFS and OS in most subgroups (Fig. S4).

**Table 2:** Univariate and multivariate Cox regression analysis of clinicopathological characteristics associated with disease-free survival in patients with colorectal cancer.

Clinicopathological characteristics	Disease-free survival			
	Univariate analysis		Multivariate analysis	
	HR (95%CI)	*P* value	HR (95%CI)	P value
Age	1.356 (1.135–1.619)	0.001	1.508 (1.256—1.810)	< 0.001
T stage (T3–4)	2.034 (1.617–2.558)	< 0.001	1.481 (1.159—1.892)	0.002
N stage				
N0	Ref			
N1	1.798 (1.460–2.214)	< 0.001	1.586 (1.280—1.967)	< 0.001
N2	4.048 (3.250–5.042)	< 0.001	3.262 (2.568—4.143)	< 0.001
Perineural invasion (Positive)	1.678 (1.292–2.179)	< 0.001	0.993 (0.742—1.328)	0.960
Vascular invasion (Positive)	1.995 (1.619–2.457)	< 0.001	1.426 (1.124—1.809)	0.003
Macroscopic type				
Protrude type	Ref			
Infiltrating type	1.539 (1.091–2.170)	0.014	1.447 (1.024—2.045)	0.036
Ulcerative type	1.387 (1.119–1.719)	0.003	1.153 (0.925—1.437)	0.205
Differentiation (High-medium)	0.699 (0.548–0.893)	0.004	0.865 (0.673—1.112)	0.256
Tumor location (Colon cancer)	0.800 (0.670–0.956)	0.014	0.783 (0.653—0.939)	0.008
CIPI (High)	1.694 (1.420–2.021)	< 0.001	1.443 (1.203—1.730)	< 0.001

*CRC* colorectal cancer; *BMI* body mass index; *CIPI* cancer-inflammation prognostic index.

**Table 3:** Univariate and multivariate Cox regression analysis of clinicopathological characteristics associated with overall survival in patients with colorectal cancer.

Clinicopathological characteristics	Overall survival			
	Univariate analysis		Multivariate analysis	
	HR (95%CI)	*P* value	HR (95%CI)	*P* value
Age	1.453 (1.206–1.749)	< 0.001	1.602 (1.323—1.939)	< 0.001
T stage (T3–4)	2.129 (1.668–2.717)	< 0.001	1.464 (1.129—1.900)	0.004
N stage				
N0	Ref		Ref	
N1	1.805 (1.452–2.244)	< 0.001	1.586 (1.266—1.987)	< 0.001
N2	4.144 (3.303–5.199)	< 0.001	3.416 (2.672—4.368)	< 0.001
Perineural invasion (Positive)	1.633 (1.244–2.143)	< 0.001	0.953 (0.703—1.292)	0.757
Vascular invasion (Positive)	2.024 (1.632–2.509)	< 0.001	1.415 (1.107—1.808)	0.006
Macroscopic type				
Protrude type	Ref		Ref	
Infiltrating type	1.535 (1.071–2.202)	0.02	1.468 (1.022—2.108)	0.038
Ulcerative type	1.406 (1.122–1.761)	0.003	1.171 (0.929—1.475)	0.181
Differentiation (High-medium)	0.643 (0.502–0.824)	< 0.001	0.800 (0.620—1.032)	0.086
Tumor size	1.218 (1.014–1.464)	0.035	1.073 (0.887—1.297)	0.468
CIPI (High)	1.780 (1.481–2.139)	< 0.001	1.442 (1.189—1.749)	< 0.001

*CRC* colorectal cancer; *BMI* body mass index; *CIPI* cancer-inflammation prognostic index.

### CIPI-based prognostic nomograms and ungrouped prediction validation

**Figure 4:**
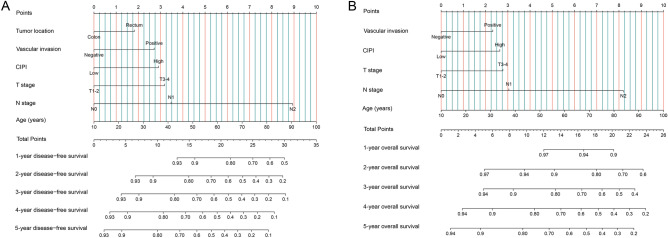
Construction the CIPI-based prognostic nomograms in CRC patients. *Notes* (**A**) The disease-free survival nomogram; (**B**) The overall survival nomogram.

We established a nomogram to predict the 1- to 5 years DFS prognosis, including independent prognostic factors determined by multivariate Cox regression analysis of DFS (Fig. 4A). This nomogram revealed an association between an increased prediction score and increasing age, a tumour location in the rectum, the occurrence of vascular invasion, an increase in the CIPI, progression of the T stage, and progression of the N stage, indicating that the risk of DFS mortality also increased. The C-index value and calibration curve were used to evaluate the discriminatory ability of the nomogram. The C-index of the nomogram was 0.683 (95% CI 0.659–0.707). The 3- and 5 years calibration plots showed good agreement between the predicted and observed values (Fig. S5A, B).

Simultaneously, we included all independent indicators in the OS multivariate analysis to construct the OS nomogram, which was used to predict the 1 to 5 years risk of OS mortality in patients with CRC (Fig. 4B). The OS nomogram included age, T and N stages, vascular invasion, and the CIPI. The greater the nomogram score, the worse the clinical prognosis of patients with CRC. The C-index of the OS nomogram was 0.687 (95% CI 0.662–0.712). The calibration plots of the OS nomogram displayed bare deviations from the 45-degree diagonal reference line, which showed the best agreement between the observed survival and survival predicted by the OS nomogram (Fig. S5C,D).

In addition, we compared the prognoses of patients with CRC predicted by the TNM staging system using the ROC curve. For DFS, the TNM staging system had a lower AUC than the DFS nomogram (3 years AUC: 0.670 vs. 0.718; 3 years AUC: 0.658 vs. 0.715) (Fig. S6A,B). We also found that the AUC of the OS nomogram was better than that of the TNM staging system in predicting the 3- and 5 years OS (3 year AUC: 0.667 vs. 0.720; 5 years AUC: 0.657 vs. 0.715) (Fig. S6C,D). Comparison of the clinical utility of our prognostic nomogram with that of the traditional TNM staging system using the DCA showed that when the threshold probability of 3 years DFS and 5 years DFS was greater than 12%, the DFS nomogram demonstrated superior accuracy to that of the TNM staging system. Similar results were observed for the OS nomogram. When the threshold probability of OS for 3 and 5 years was predicted to be greater than 12%, the OS nomogram showed better accuracy than TNM staging (Fig. S7).

### Internal subgroup validation performance

**Figure 5:**
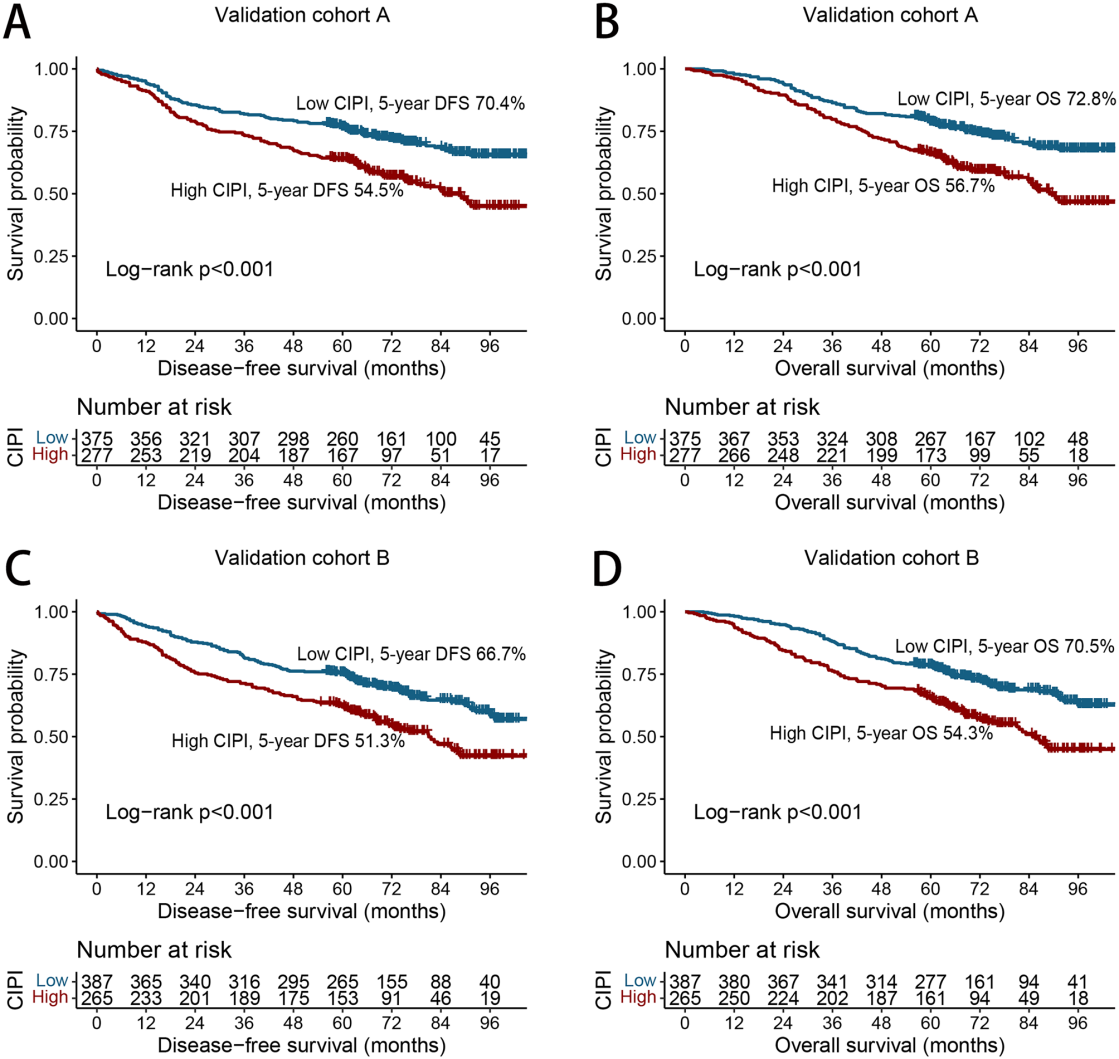
Kaplan–Meier curve of CIPI in patients with colorectal cancer at internal validation cohorts. *Notes* (**A**) Disease-free survival at validation cohort A; (**B**) Overall survival at validation cohort A; (**C**) Disease-free survival at validation cohort B; (**D**) Overall survival at validation cohort B.

Patients were divided randomly into validation cohorts A (n = 652) and B (n = 652) in a 1:1 ratio. Table S1 lists the clinicopathological characteristics of the patients with CRC in the two cohorts. No statistically significant difference was found between the two cohorts for the clinicopathological characteristics. In validation cohort A, the DFS (54.5% vs. 70.4%, *p* < 0.036) (Fig. 5A) and OS (56.7% vs. 72.8%, *p* < 0.001) (Fig. 5B) of patients in the high-CIPI group were significantly less than those in the low-CIPI group. In validation cohort B, the CIPI effectively classified the prognosis of DFS (51.3% vs. 66.7%, *p* < 0.001) (Fig. 5C) and OS (54.3% vs. 70.5%, *p* < 0.001) (Fig. 5D) for patients with CRC.

The C-indices of the DFS and OS nomograms in validation cohort A were 0.689 (95% CI 0.655–0.723) and 0.685 (95% CI 0.651–0.719), respectively. In validation cohort B, the C-indices of the DFS and OS nomograms were 0.681 (95% CI 0.647–0.715) and 0.694 (95% CI 0.658–0.730), respectively. Furthermore, the 3-year and 5-year DFS/OS calibration charts showed that the actual observed survival was most consistent with the survival predicted by nomograms in validation cohorts A (Fig. S8A,B) and B (Fig. S8C,D).

## Discussion

The CIPI is a newly developed, simple, and effective prognostic indicator. Based on neutrophil, lymphocyte, and CEA levels, Su et al.^19^ developed the CIPI in 2021 to predict survival in patients with metastatic CRC treated with regorafenib. A subsequent study by You et al.^22^ also showed that the CIPI is a reliable and easy-to-use clinical factor for predicting CRC outcomes. However, few studies are available on the prognostic value of the CIPI in patients with CRC. This study demonstrated that the CIPI is a promising novel tool for predicting recurrence and survival in patients with stage I–III CRC. We found that the optimal cut-off for the CIPI in patients with stage I–III CRC was 11. The CIPI can effectively stratify the prognoses of patients with CRC. The recurrence and mortality rates of the high-CIPI group were significantly greater than those of the low-CIPI group. The CIPI can also serve as an effective auxiliary tool to further distinguish the prognoses of CRC patients at the same pathological stage, especially for stages II and III. In addition, compared to other commonly used prognostic indicators, CIPI demonstrates superior predictive accuracy for CRC prognosis. Subsequently, internal validation demonstrated that the CIPI has a wide range of applications for predicting DFS and OS in patients with CRC. In addition, based on the independent prognostic factors identified in the multivariate analysis, we constructed CIPI-based nomograms to predict the 1–5 years DFS/OS and OS in patients with CRC. Compared with traditional TNM staging, nomograms have a better predictive performance. These results suggest that the CIPI is a useful biomarker for predicting the prognoses of CRC patients. The CIPI prognostic nomograms were beneficial for prognostic prediction, efficacy evaluation, and treatment formulation in CRC patients.

Systemic inflammation is caused by complex host–tumour interactions and plays a crucial role in cancer development; it is considered the seventh sign of cancer and is involved in cancer development, proliferation, metastasis, senescence, and apoptosis^8,23^. Neutrophils and lymphocytes are important cellular components involved in systemic inflammation. Lymphocytes play an important role in cancer immune monitoring by inhibiting tumour cell proliferation, inhibiting metastasis through cytokine production, and inducing cytotoxic cell death^24,25^. As the first line of defence in the inflammatory response, neutrophils may suppress the immune system by inhibiting the cytolytic activity of immune cells such as lymphocytes, activated T cells, and natural killer cells. They can also secrete cytokines and chemokines that create a tumour microenvironment suitable for tumour cell proliferation, invasion, and micro angiogenesis, thus promoting tumour development and progression^26,27^. The combination of blood-derived neutrophils and lymphocytes is reported to be an effective indicator of systemic inflammation and an effective predictor of the prognosis of CRC patients^21,28,29^. Serum CEA is a useful marker of CRC, and its increase is associated with a poor prognosis for patients with CRC. Moretto et al.^15^ demonstrated that CEA levels can accurately predict the response after the end of first-line induction therapy. Kim et al.^18^ found that preoperative serum CEA levels were an independent prognostic factor for DFS and OS after radical resection and adjuvant chemotherapy in stage III colon cancer. Lalosevic et al.^17^ also found that the serum CEA level could be used as a diagnostic factor for CRC severity, specifically indicating the occurrence of CRC metastasis. One possible explanation for the association between the CIPI and CRC prognoses is that the CIPI accounts for a combination of systemic inflammation and tumour load in CRC patients. Patients with high CIPI values, which indicate great tumour aggressiveness and high tumour-related inflammation, may be considered for closer follow-up monitoring or even more-aggressive treatment strategies to improve their prognosis.

Elevated CIPI values may indicate more-aggressive tumour features (advanced T stage, advanced N stage, advanced TNM stage, high CEA, and large tumour diameter) and more-severe tumour-related inflammation (high neutrophil and low lymphocyte counts). Compared with patients with a low CIPI, those with a high CIPI had significantly increased recurrence, overall death, length of stay, and hospitalisation costs. In clinical practice, the prognoses of patients vary greatly, even among those at the same pathological stage. We found that the CIPI can be used as a useful prognostic supplement for pathological staging, optimising individualised tumour risk stratification in CRC patients. It is worth noting that with the aggravation of the pathological stage, the CIPI performs better in the prognosis stratification of patients with CRC. This finding may be due to the greater tumour load and inflammatory burden of patients with advanced cancer, which is more prone to tumour-cell proliferation and invasion and tumour neovascularisation, leading to changes in the proportion of CEA, neutrophils, and lymphocytes. These results suggest that the CIPI may be more suitable for the prognostic assessment in patients with advanced-stage CRC.

For intuitive use in clinical work, we have constructed novel and effective CIPI-based nomograms to facilitate individual prognosis prediction and clinical treatment monitoring decisions for patients with CRC. These nomograms have the advantage of integrating individual profiles, tumour characteristics, and serum inflammatory markers, providing favourable discrimination and calibration values. Subsequently, we confirmed that these nomograms have good application prospects through internal queues. Compared with traditional TNM staging, CIPI-based prognostic nomograms have better resolution and accuracy in predicting 3- and 5 years DFS and OS in patients with CRC. We believe that these nomograms can help clinicians directly quantify the prognostic risk of CRC, thus making it easier to develop appropriate treatment strategies for CRC patients.

This study had several limitations. First, it is important to note that our study is based on a population cohort, and therefore, further experimental validation is needed to confirm the utility of CIPI as a prognostic marker for CRC. selection bias cannot be ignored due to the study’s retrospective nature. Second, because this study included only CIPI data at one time-point before the operation, we could not explore the effect of the CIPI trajectory on the prognoses of patients with CRC. Ultimately, whether CIPI-based nomograms can be applied directly to other populations is unclear, and their universality requires further external validation and prospective evaluation.

## Conclusion

The serum CIPI is an effective and easy-to-use clinical tool for predicting the recurrence and overall mortality of patients with stage I–III CRC and can be a useful prognostic supplement for pathological staging, especially for patients at advanced stages. In addition, the CIPI-based nomograms have good predictive accuracy, can help clinicians quantify the prognostic risk of CRC patients, and can provide a basis for optimising treatment decisions for CRC patients.

### Supplementary Information

Supplementary Information.

## Data Availability

All data generated or analyzed during this study are included in this article and its supplementary material files. Further enquiries can be directed to the corresponding author.
