# Supplementary material for: The cancer inflammation prognostic index is a valuable biomarker for predicting the survival of patients with stage I–III colorectal cancer
Source: Sci Rep. 2023 Oct 23;13:18080. doi: 10.1038/s41598-023-45550-0 (PMC10593829; doi:10.1038/s41598-023-45550-0)
Supplement: Supplementary file 1 — Supplementary Information. [file 41598_2023_45550_MOESM1_ESM.docx]

**Supplemental material**

**Figure S1.** The optimal threshold of CIPI in patients with colorectal cancer.

**
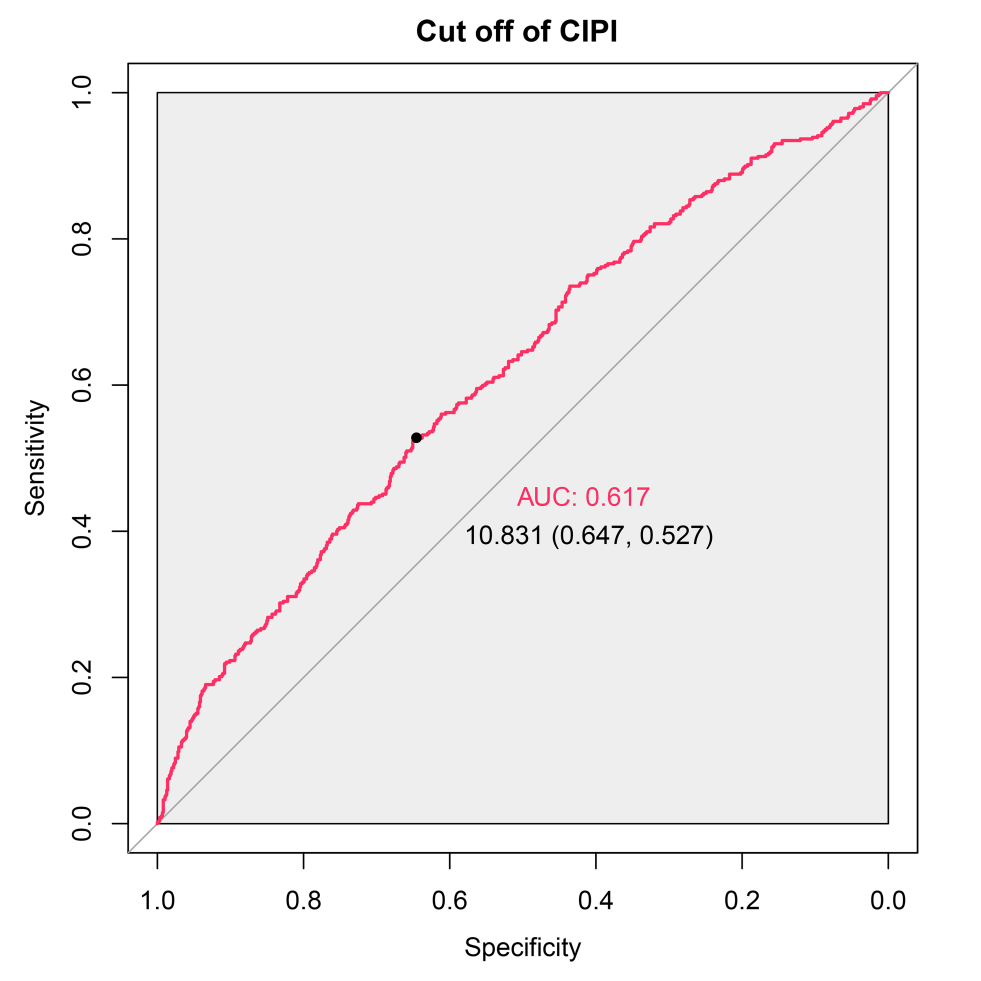
**

**Figure S2.** Median CIPI of patients alive and with recurrence or death during follow-up

**
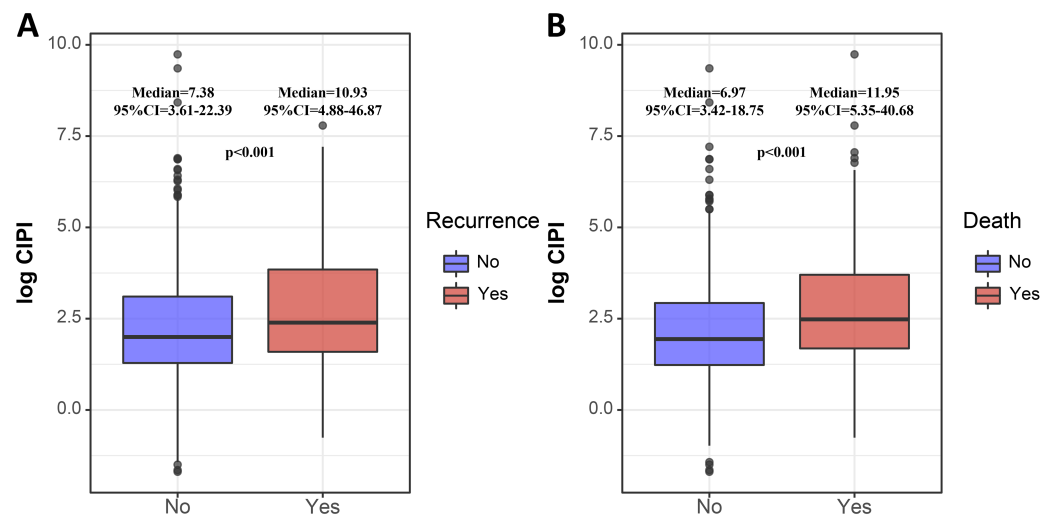
**

**Figure S3.** Stratified Kaplan-Meier curve of CIPI based on TNM stage in patients with colorectal cancer.


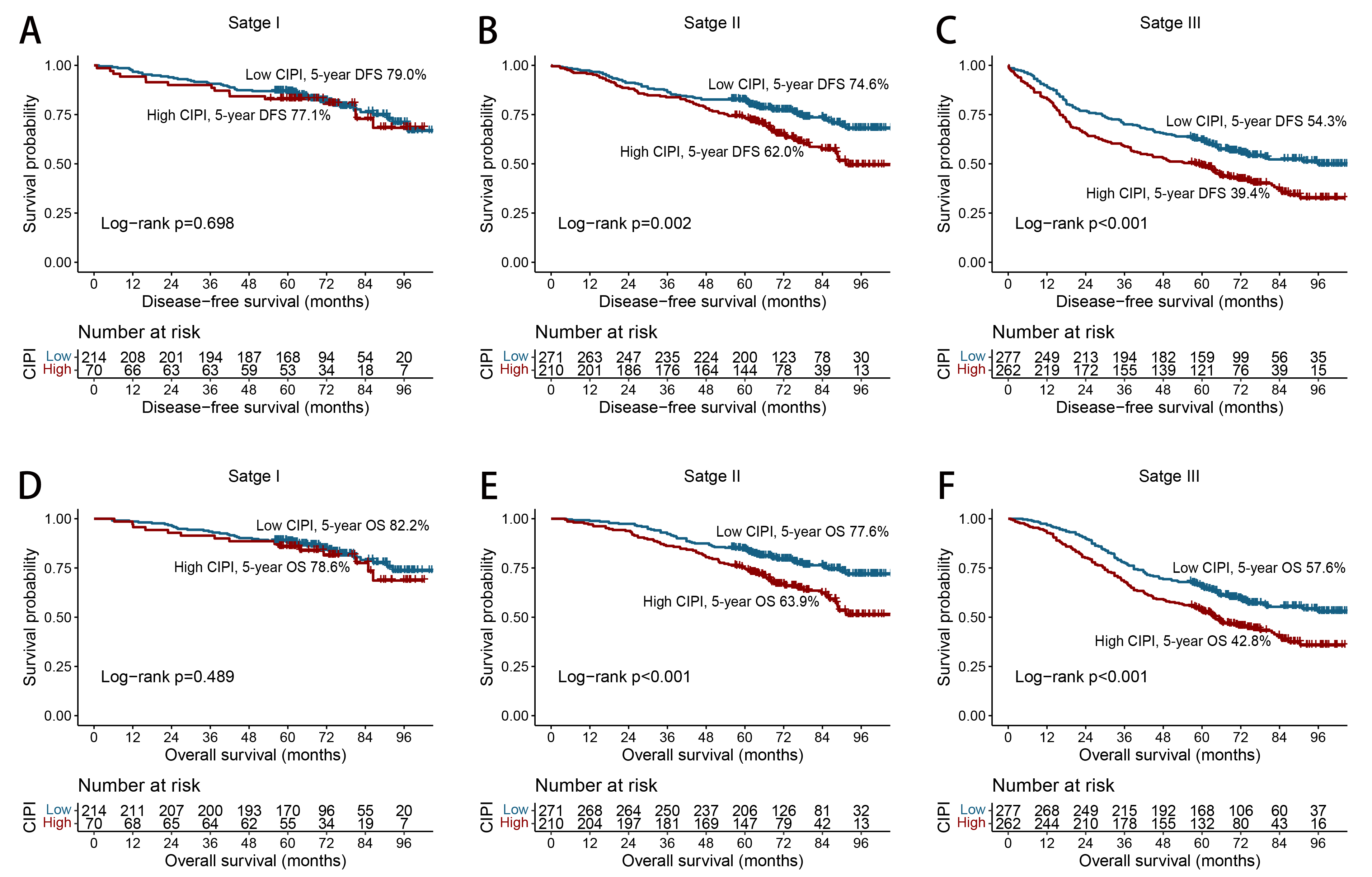


**Notes:** A, Disease-free survival of TNM I stage; B, Disease-free of TNM II stage; C, Disease-free of TNM III stage; D, Overall survival of TNM I stage; E, Overall survival of TNM II stage; F, Overall survival of TNM III stage.

**Figure S4.** The association between CIPI and hazard ratio of OS and PFS in various subgroups.


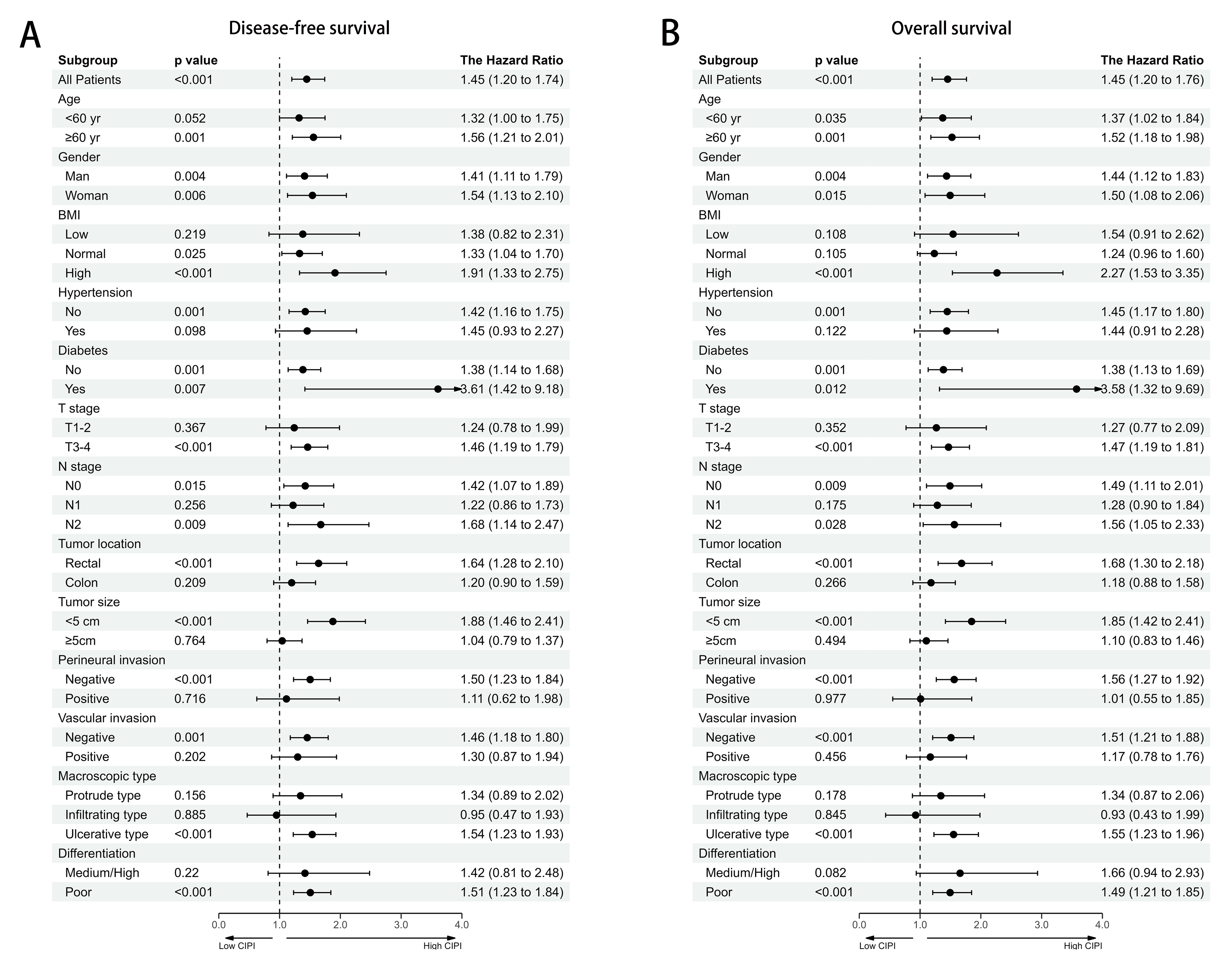


**Notes:** A, Disease-free survival, B, Overall survival

**Figure S5.** The calibration curve of the DFS and OS nomograms in patients with colorectal cancer.

**
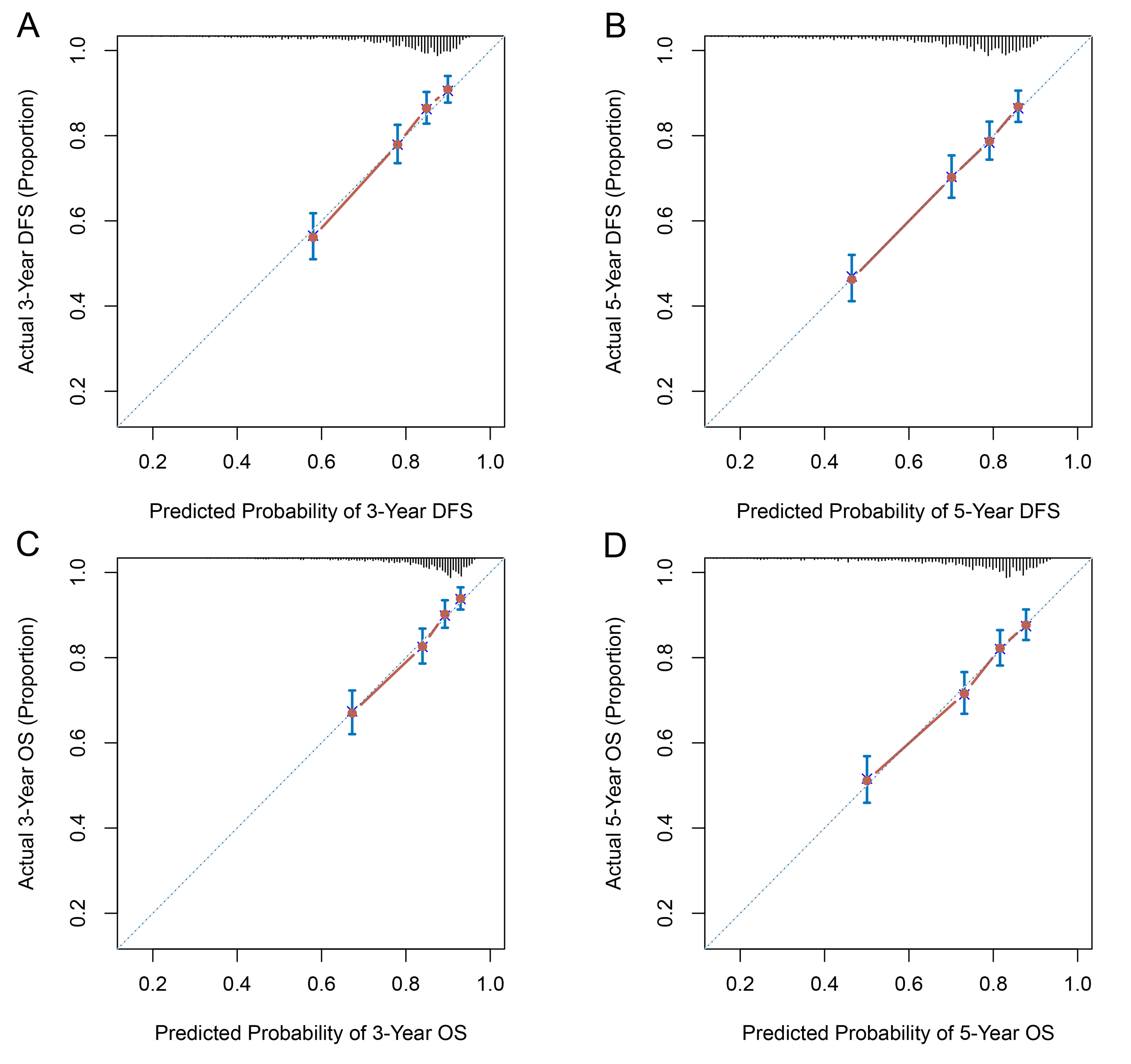
**

**Notes:** A, 3-year DFS; B, 5-year DFS; C, 3-year OS; D, 5-year OS.

**Figure S6.** Comparison of the ability of the novel prognostic nomograms and TNM stage in predicting PFS and OS of patients with colorectal cancer.

**
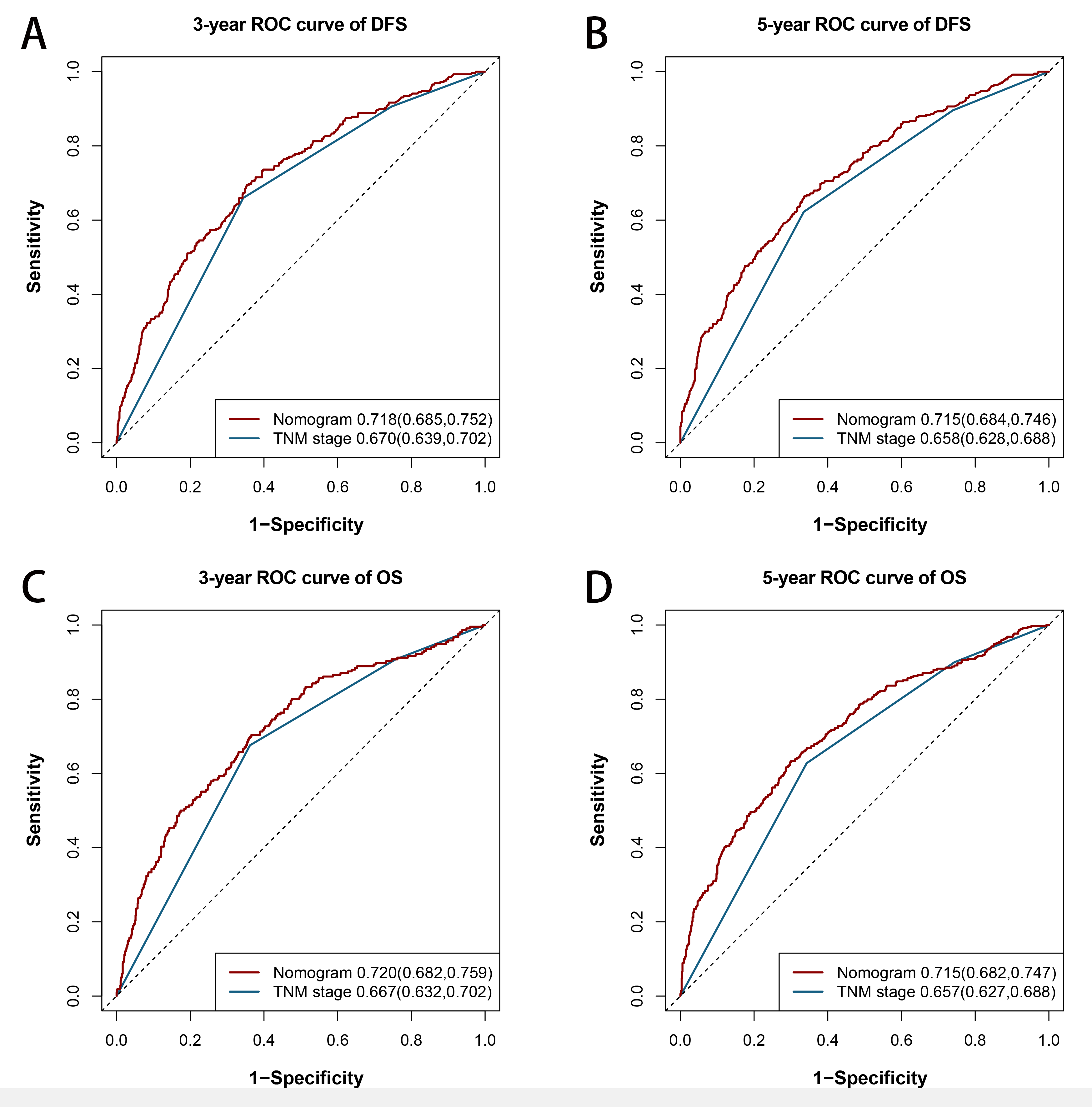
**

Notes: A, 3-year DFS; B, 5-year DFS; C, 3-year OS; D, 5-year OS.

**Figure S7.** Comparison of the decision curve of prognostic nomogram and traditional TNM stage in predicting prognosis at 3-year and 5-year.


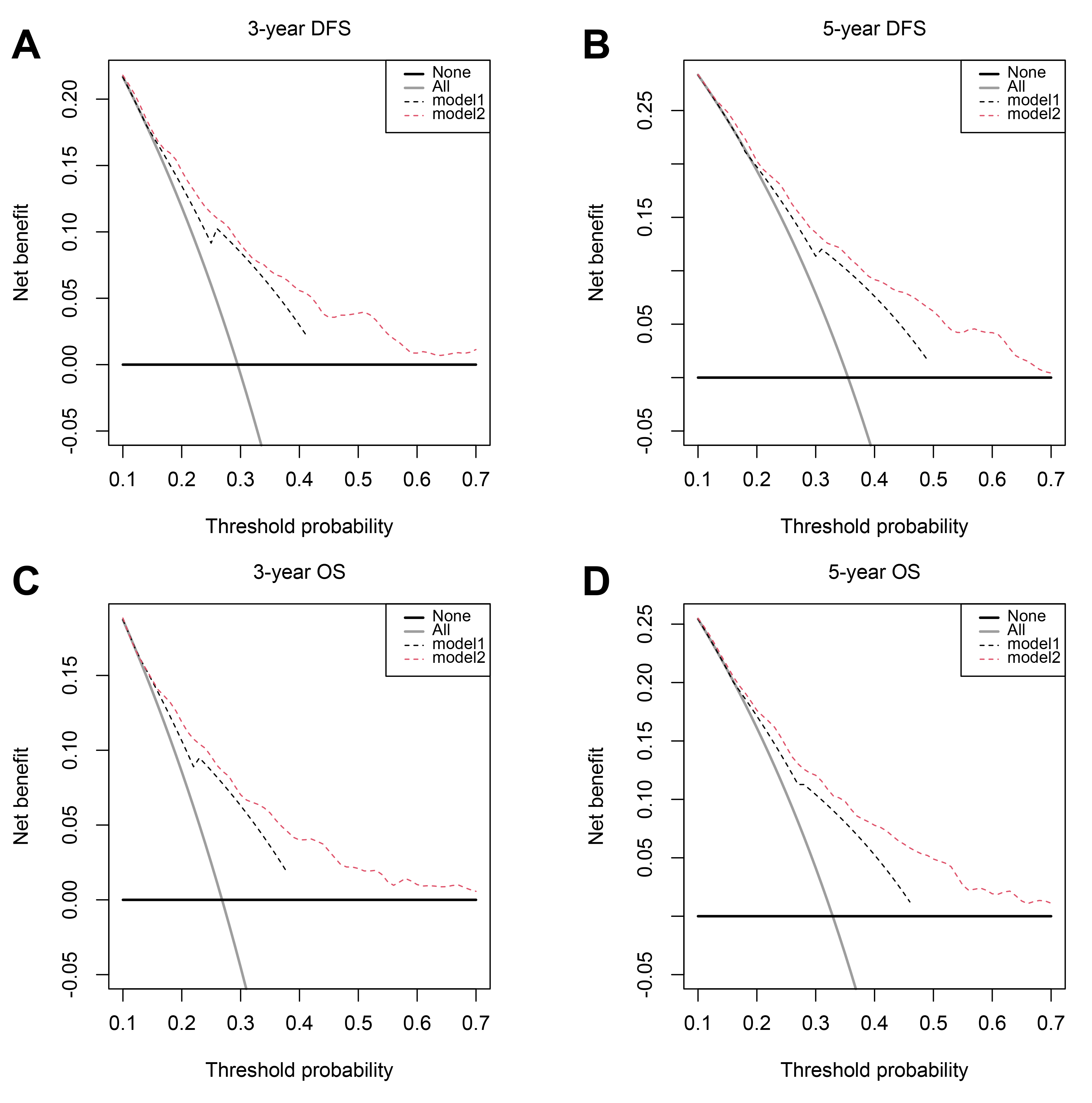


**Notes:** A, 3-year DFS; B, 5-year DFS; C, 3-year OS; D, 5-year OS.

**Figure S8.** The calibration curve at randomize internal validation cohorts.

**
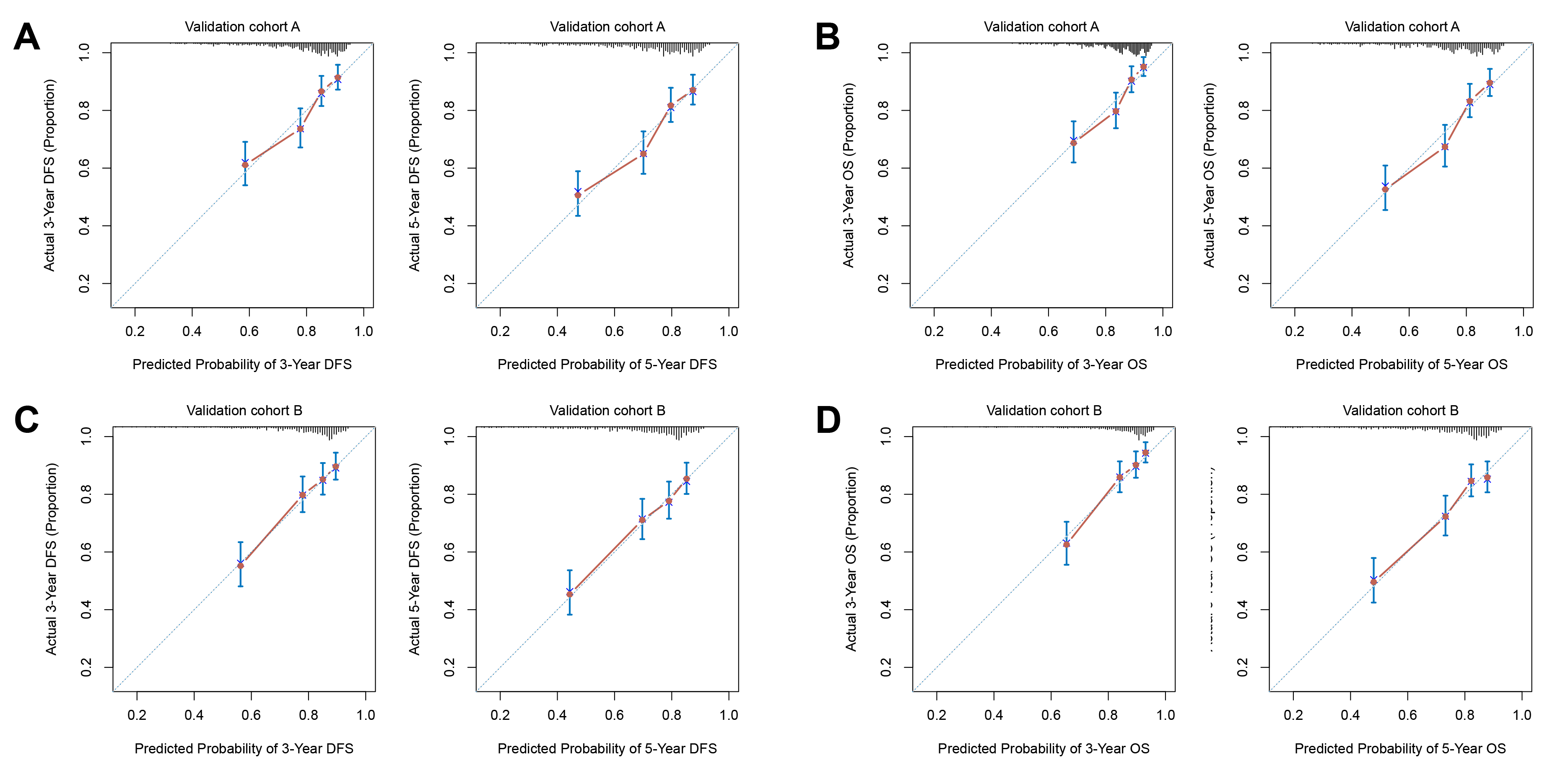
**

**Notes:** A, DFS at validation cohort A; B,OS at validation cohort B; C, DFS at validation cohort B; D, OS at validation cohort B.

**Table S1.** The clinicopathological characteristics of two validation cohorts in patients with colorectal cancer.

| Clinicopathological characteristics | Validation cohort A  (n = 652) | Validation cohort B  (n = 652) | P value |
| --- | --- | --- | --- |
| Sex(Man) | 406 (62.3) | 415 (63.7) | 0.646 |
| Age (mean (SD)) | 58.29 (13.05) | 58.34 (12.96) | 0.937 |
| BMI (median [IQR]) | 22.15 (19.98, 24.61) | 22.05 (20.09, 24.22) | 0.507 |
| Hypertension (Yes) | 114 (17.5) | 104 (16.0) | 0.504 |
| Diabetes (Yes) | 39 (6.0) | 43 (6.6) | 0.732 |
| T stage (T3-4) | 482 (73.9) | 458 (70.2) | 0.156 |
| N stage |  |  | 0.464 |
| N0 | 133 (20.4) | 151 (23.2) |  |
| N1 | 242 (37.1) | 238 (36.5) |  |
| N2 | 277 (42.5) | 263 (40.3) |  |
| TNM stage (III-IV) |  |  | 0.464 |
| Stage I | 133 (20.4) | 151 (23.2) |  |
| Stage II | 242 (37.1) | 238 (36.5) |  |
| Stage III | 277 (42.5) | 263 (40.3) |  |
| Perineural invasion (Yes) | 58 (8.9) | 64 (9.8) | 0.634 |
| Vascular invasion (Yes) | 90 (13.8) | 118 (18.1) | 0.041 |
| Macroscopic type |  |  | 0.008 |
| Protrude type | 164 (25.2) | 211 (32.4) |  |
| Infiltrating type | 59 (9.0) | 43 (6.6) |  |
| Ulcerative type | 429 (65.8) | 398 (61.0) |  |
| Differentiation (Poor) | 89 (13.7) | 79 (12.1) | 0.457 |
| Tumor location (Rectal) | 342 (52.5) | 345 (52.9) | 0.912 |
| Tumor size (median [IQR]) | 4.50 (3.50, 5.50) | 4.50 (3.50, 6.00) | 0.576 |
| CEA (High) | 238 (36.5) | 256 (39.3) | 0.332 |
| Neutrophil | 3.85 (3.04, 5.01) | 3.74 (2.97, 4.76) | 0.065 |
| Lymphocyte | 1.80 (1.39, 2.20) | 1.74 (1.42, 2.19) | 0.289 |
| Radiotherapy (Yes) | 57 ( 8.7) | 67 (10.3) | 0.396 |
| Chemotherapy (Yes) | 281 (43.1) | 300 (46.0) | 0.316 |
| Death (Yes) | 222 (34.0) | 235 (36.0) | 0.486 |
| Length of stay (median [IQR]) | 17.00 (11.00, 21.00) | 16.00 (11.00, 20.00) | 0.259 |
| Hospitalization cost (median [IQR]) | 49457.30 (44835.22, 55753.53) | 49211.90 (44522.34, 55607.45) | 0.546 |

**Table Note:** CRC, colorectal cancer; BMI, body mass index.
